# Promoting Identification and Use of Aid Resources by Caregivers of Seniors: Co-Design of an Electronic Health Tool

**DOI:** 10.2196/12314

**Published:** 2019-08-22

**Authors:** Dominique Giroux, Mélanie Tremblay, Karine Latulippe, Véronique Provencher, Valérie Poulin, Anik Giguere, Véronique Dubé, Andrée Sévigny, Manon Guay, Sophie Ethier, Maude Carignan

**Affiliations:** 1 Department of Rehabilitation Université Laval Québec, QC Canada; 2 Center of Excellence on Aging Quebec Québec, QC Canada; 3 Department of Teaching and Learning Studies Université Laval Québec, QC Canada; 4 School of Rehabilitation Université de Sherbrooke Sherbrooke, QC Canada; 5 Center of Research on Aging Centre intégré universitaire de santé et de services sociaux de l’Estrie Centre hospitalier universitaire de Sherbrooke Sherbrooke, QC Canada; 6 Université du Québec in Trois-Rivières Trois-Rivières, QC Canada; 7 Interdisciplinary Center for Research in Rehabilitation and Social Integration Université Laval Québec, QC Canada; 8 Department of Family Medicine and Emergency Medicine Université Laval Québec, QC Canada; 9 Faculty of Nursing University of Montreal Montreal, QC Canada; 10 Research Centre of the University Hospital of Montreal Montréal, QC Canada; 11 School of Social Work and Criminology Université Laval Québec, QC Canada

**Keywords:** caregivers, aged, help-seeking behavior, community-based participatory research, eHealth, telemedicine

## Abstract

**Background:**

The importance of supporting caregivers is recognized in home care for older persons, and facilitating their help-seeking process is a way to meet that need. The use of electronic health (eHealth) is a potentially promising solution to facilitate caregivers’ help-seeking process.

**Objective:**

The aim of this research was to develop, in partnership with community organizations, health and social service professionals and caregivers, an eHealth tool promoting the earlier identification of needs of older persons and an optimal use of available resources.

**Methods:**

To design the tool, 8 co-design sessions (CoDs) were conducted and 3 advisory committees were created (in 11 regions) in Quebec between May 2017 and May 2018. A variety of methods were used, including the sorting method, the use of personas, eHealth tool analysis, brainstorming, sketching, prototyping, and pretesting.

**Results:**

A total of 74 co-designers (women n=64 and men n=10) were recruited to participate in the CoDs or the advisory committees. This number allowed for the identification of needs to which the tool must respond and for the identification of its requirements (functionalities and content), as well as for the development of the information architecture. Throughout the study, adjustments were made to the planning of CoD, notably because certain steps required more sessions than expected. Among others, this was true for the identification of functionalities.

**Conclusions:**

This study led to the development of an eHealth tool for caregivers of functionally dependent older persons to help them identify their needs and the resources available to meet them.

**International Registered Report Identifier (IRRID):**

RR2-10.2196/11634

## Introduction

### Background

It is recognized that aging of the population has an impact on health and social service professionals (HSSPs) who provide care and services to these individuals. This reality, added to a recognition of the benefits of keeping seniors at home [1,2], has resulted in a reorganization of services in Quebec where home care is now promoted [3]. This choice has an impact on caregivers, who are often asked to contribute to home care for older persons with decreasing independence, both physical and psychological [4-6]. Although this role can be rewarding, *l’Appui pour les proches aidants d’aînés*, a nonprofit organization in Quebec that supports caregivers, reveals that 99% (3771/3809) of participants in their study reported a negative impact of caregiving on their health [5]. They report anxiety or anguish (37% 1409/3809), fatigue (32% 1219/3809), and sleep problems (22% 838/3809) and mention needing home care (28% 1067/3809), professional help (26% 990/3809), respite (23% 876/3809), information and advice (12% 457/3809), and support (11% 419/3809) [5]. It is important to note that although nearly one in 4 participants report needing respite, 94% (3581/3809) say they never use these services [5].

So, the importance of assisting caregivers is recognized and, in response, many support programs, resources, and services are offered by health and social service facilities and by community-based and private organizations [6]. There are a number of benefits to using these services, both for the caregiver and the person with decreasing independence [6]. However, it appears that they are still less used. Some tools exist in Quebec to facilitate caregivers’ help-seeking process but most are intended for HSSPs and not specifically for caregivers. Moreover, it appears that despite the existence of these tools, the available resources are not widely known and they are seldom used by caregivers.

A pilot study conducted by Latulippe et al [7] highlights the factors influencing caregivers’ help-seeking process. This study reveals that they need effective tools early in the process of the loss of independence to help them identify the appropriate resources to meet their needs and those of the older person they are helping. It is often after the first signs of exhaustion that caregivers undertake the help-seeking process, but it is difficult for them to know the most appropriate resource for their situation without the assistance of HSSPs [8,9]. The use of electronic health (eHealth) is a potentially promising solution to facilitate caregivers’ help-seeking process [10]. Thus, the goal of this research was to develop, in partnership with community organizations, HSSPs, and caregivers, an eHealth tool promoting an earlier identification of the needs of functionally dependent seniors and an optimal use of available resources.

### Conceptual Framework

**Figure 1:**
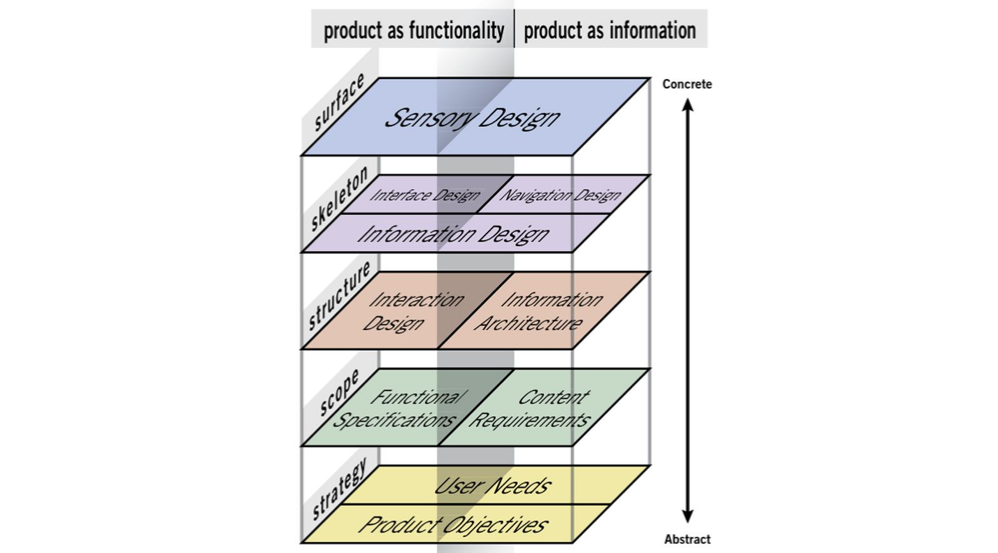
Elements of user experience (Garrett 2011).

To develop this eHealth tool, we followed a user experience (UX) perspective. Using a UX perspective for the design of a technology involves going beyond instrumental need and acknowledging the use of this technology as a “subjective, situated, complex and dynamic encounter,” considering the user’s internal state, the characteristics of the product design and the context of interaction with the product [11]. We used the *Elements of User Experience* UX framework (Figure 1), which proposes 5 steps for the development of user-centered technologies: (1) identification of the strategy (product objectives and user’s needs), (2) identification of the scope (functional specifications and required content), (3) development of the structure (interaction design and information architecture), (4) creation of the skeleton (interface, information, and navigation design), and (5) creation of the surface (sensory design) [12].

### Objectives of This Paper

**Figure 2:**
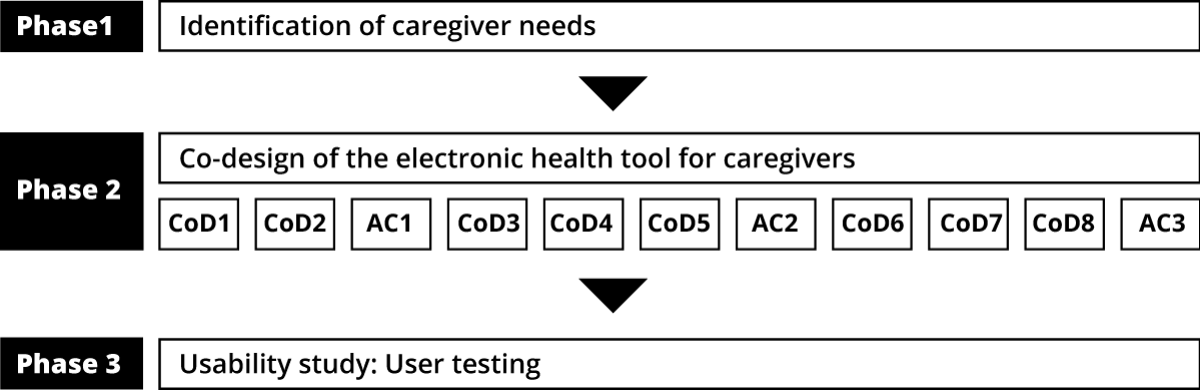
Design phase of the entire project and steps involved in this publication (in bold). CoD: co-design session; AC: advisory committee session.

The protocol of this project presenting the details of the methodology has been published in the *Journal of Medical Internet Research* protocols [13]. The results of phase 2 of this research are now presented in 3 different papers. The first focuses on identifying needs as the first step in co-design. The second concerns the development of the functionalities and contents of the tool. The purpose of the third article, this paper, was to present the whole process of phase 2: the development of an eHealth tool for caregivers using a co-design approach. It also aims to explain the differences between what was planned and what was achieved, to present the tool developed, and to discuss the benefits and challenges of using a co-design approach. Figure 2 illustrates where this paper is situated in the entire process of the study.

## Methods

### Study Design

A method based on a co-design participatory approach was used to achieve the objectives of this study. According to Harder et al [14], the co-design approach is different from the positivist perspective as participants are not studied objectively. From a co-design perspective, the distinctions between researcher, practitioner, and user are blurred. At the end of the level of participation spectrum, engagement of the participant is described as “full partnership” or “learning as one”[…] [14]. On the basis of a literature review of 10 years (2002-2012) of participatory design (PD) research, Halskov and Hansen present the fundamental aspects of PD [15]—(1) politics: people who are affected by a decision should have an opportunity to influence it, (2) people: people play critical roles in design as experts in their own lives, (3) context: the use situation is the fundamental starting point for the design process, (4) methods: methods are means for users to gain influence in design processes, and (5) product: the goal of participation is to design alternatives, improving the quality of life.

According to this approach, the actors directly concerned by the project objectives, here the caregivers themselves, as well as the community organizations and HSSPs providing care and services, were included at each stage of the study, not as participants but as co-designers [16]. This approach ensures that the tool meets the user needs for an eHealth tool [17].

To apply this approach, 8 co-design sessions (CoDs) and 3 advisory committee sessions (ACs) were planned (Figure 2). The CoDs, lasting 3 hours, consisted of following the steps of Garrett’s model (Figure 1). The ACs were also 3 hours long, and the role of these committees was to guide the progression of the tool to ensure continuity between the CoDs and coherence between the decisions taken by the co-designers participating in different sessions [13].

To optimize the tool, we considered the factors contributing to reducing social health inequalities (engagement of future users in the co-design, the help-seeking process, access to eHealth technologies, knowledge related to the utilization of eHealth technologies, eHealth literacy and cultural competency, and learning capacity) throughout the development of the eHealth tool [18]. This aspect is the subject of the thesis of one doctoral student who is part of the research team and will, therefore, not be discussed in the context of this article. We also considered the intrinsic experience of people who participated as co-designers during the CoDs and ACs. This aspect is the subject of the thesis of another doctoral student who is also on the research team. Their publications will come later.

As mentioned earlier, a more detailed description of the method (participants and selection criteria, recruitment, content planned for each CoD, data collection, and ethical considerations) can be found in the published protocol of the study [13]. We still offer a summary for an overall assessment of the process.

### Recruitment

The sampling strategy was based on the importance of including all potential users in designing the tool. Therefore, co-designers were recruited from 3 categories of users: caregivers, service providers from community settings, and professionals from the health and social services network. The advisory committee included researchers (VP, VP, VD, and SE), caregivers, community workers, and HSSPs. The research team included a Doctor of Philosophy (PhD) student and UX expert (MT), a PhD student (KL) working on the factors reducing social health inequalities, a research professional who is an anthropologist and trained in qualitative research (MC), and the project director (DG).

This study was a multicentric project. To ensure a representative sampling of the different situations in Quebec regions, recruitment covered 11 regions of Quebec (including rural and urban areas) between May 2017 and May 2018. To recruit co-designers, the home care and elderly care management of the 11 Integrated Health and Social Service Centres (CISSS) were contacted to recruit 2 HSSPs per CISSS. In addition, these workers were asked to recruit caregivers using their services. Members of community organizations were recruited through direct contact via phone or email. They were also asked to publicize our recruitment announcement among caregivers attending their institution and activities. Finally, recruitment announcements for caregivers were posted in 30 family medicine groups throughout the province.

The study received ethical approval from the *comité d’éthique du CIUSSS Capitale Nationale* (2016-2017-10MP), and informed consent was obtained from each participant. Participants also received a symbolic compensation amount (Can $20) to cover potential fees for travel and parking.

### Data Collection

A variety of methods were used to promote participation of all co-designers in the process throughout the project’s evolution. Sometimes the activities were carried out in a large group (project presentations, the sorting method, plenary sessions, brainstorming, and the conclusions) and sometimes in subgroups (prototyping, eHealth Tool comparative analysis, sketching, and pretesting). The subgroups were divided in a mixed way or by type of co-designers (caregivers, HSSPs, or community workers). Mixed subgroups were used when we wanted to cross perspectives, whereas division by type of co-designer was used when we wished to highlight the perception of caregivers. For each subgroup, a moderator (a member of the team for each subgroup) monitored the conduct of the activity and the role of each participant.

As illustrated in Figure 2, the CoDs were interspersed with the ACs (September 2017, December 2017, and June 2018). Members of the advisory committee did not intervene directly in the CoD. However, results collected during the CoDs were reported to the advisory committee when decisions had to be taken or when co-designers differed. These decisions were made by reaching a consensus among the committee members.

The data were obtained via the notes taken by moderators during and after the working sessions, any artifacts produced, and a synthesis of audio recordings. The role of the research team was very important in this study as they were also acting as co-designers, according to the co-design study plan [17]. Each member of the research team participated in data collection and worked in partnership with other co-designers at every step of the design process.

### Data Analysis

For data analysis, an analytical questioning method was employed [19]. This method consists of the development of an investigative framework according to the research objective, followed by careful and repeated reading of the material under study to answer the initial questions. Therefore, the researcher questions the corpus, acquires a first-level response, and converts the answers into additional and more precise questions. Finally, by answering these newly generated questions, we obtain more detailed answers or new questions if needed.

Following the analytical questioning method, the objectives of each CoD were articulated in question form as a first step. The investigative framework for each session is detailed in Table 1.

**Table 1:** Investigative framework.

Analytical questions	Co-design (CoD) and advisory committee (AC) sessions
Based on the needs identified in the pilot project, the literature and potential additions by the group, which ones should be prioritized in the design of the tool?	CoD1 and AC1
What are the general requirements (ex: I want someone to be there to answer my questions) and specific requirements to consider (ex: I want a forum)?	CoD1, AC1, CoD3, and CoD4
What does the tool need to do to meet these needs in considering the characteristics of the individuals concerned and their own experience?	CoD2
Based on the identified needs, what would this tool look like, what would it do?	AC1
What features would meet the needs and requirements of previous groups?	CoD3, CoD4, CoD5, AC2, CoD6, and CoD7
What content elements would meet the needs and requirements of previous groups?	CoD5, AC2, CoD6, and CoD7
Which architecture or structural design of the information would facilitate intuitive access to content?	CoD5, AC2, CoD6, CoD7, CoD8, and AC3
How should we interact with the site functionalities to facilitate intuitive access to content?	CoD5, AC2, CoD6, CoD7, CoD8, and AC3
What design of interface elements can facilitate interaction between the user and the functionalities, as well as movements through the architecture?	CoD7, CoD8, and AC3
How effective are the graphic processing of the elements of the interface, the visual processing of the text, the elements of the page and the navigation?	CoD8 and AC3

The research team systematically applied the analytical questioning method after every CoD and AC. Each member condensed the data (notes, artifacts, and a synthesis of audio recordings) of their subgroup and, for group activities (plenary discussions), 1 member was designated to perform the analysis. According to the investigative framework, answers to the initial questions were reported in a Microsoft Word or Excel document. Subsequently, several meetings were held to discuss the analytical results, to verify and confirm the results obtained, and to check whether the objectives were achieved or if more work was needed to reach them. This data analysis was necessary to plan the following session.

## Results

### Co-Designers’ Characteristics

A total of 74 co-designers (women n=64 and men n=10) were recruited for this project (Table 2).

We initially hoped to have co-designers with a variety of characteristics, in terms of their profession (social worker, occupational therapist, physiotherapist, doctor, and nurse), their organization (administrative agency, association, organization, and other), and their sociodemographic attributes, to ensure that the tool is developed taking into account a diversity of people [13]. This appears to have been achieved, except for gender and ethnicity. Caregivers, community workers, and HSSPs are more often female [20,21]; this reflects the reality. Furthermore, our co-designers were predominantly Caucasian.

**Table 2:** Description of co-designers.

Caregivers socio-demographic characteristics		Caregivers (n=30)	Community workers (n=26)	Health professionals (n=18)
**Sex, n**				
	Women	26	20	18
	Men	4	6	0
Age (years), range (mean)		42-88 (77.9)	24-66 (44.8)	29-53 (39.6)
**Education level, n**				
	Elementary school	1	0	0
	High school	10	1	0
	College	4	4	6
	Vocational studies	1	0	3
	University	12	21	9
	None	1	0	0
	N/M^a^	1	0	0
Age of the relative (years), range (mean)		61-96 (78.2)	—^b^	—
**Relationship to the person for whom they provide care, n**				
	Children	8	—	—
	Sibling	3	—	—
	Spouse/husband	17	—	—
	Friend	2	—	—

^a^Not mentioned.

^b^Not applicable.

### Co-Design Process

Throughout the progress of the study, adjustments were made to plan the CoD, notably because certain steps, among others the identification of functionalities, required more sessions than expected. A potential explanation for this is that the co-designers were not experts in Web design, and they had more difficulty identifying the functionalities needed to meet the targeted needs. In accordance with the design process and with the study by Garrett [12], existing tools in the same category should be analyzed. The choice was made to explore 9 existing eHealth tools (selected by considering the functionalities included to expose participants to a variety) with the co-designers to help determine which seemed relevant to meet the identified needs (CoD3). The development of content items also took longer than expected. Table 3 summarizes the planned content of the CoDs and the ACs, as described in the study by Latulippe et al [13], the content covered after the adjustments, the methods used, and the results achieved.

**Table 3:** Co-design sessions’ content.

Co-design session and content planned		Covered content	Activities and methods	Achievements
**1, 2, and 3—Strategy: user needs and product objectives**				
	Identification of content and functional requirements that must be included to meet the user’s needs	Identification of the needs; identification of tool requirements based on prioritized needs and the variables to consider; and comparison of existing tools	Sorting method; brainstorming; persona; and workshops in subgroups	Identification of 8 needs not covered by any contents or functionalities in the other tools
**4 and 5—Scope and structure: functional specifications, content requirement, interaction design, and information architecture**				
	Identification of content and functional requirements that must be included to meet the user’s needs (continued); design of information architecture to facilitate intuitive access to content; interaction design: development of the course of the application with the aim of facilitating the user’s tasks and defining how the user interacts with the functionalities of the site	Identification of content and functional requirements to meet the 8 user’s needs not covered by the other tools; choice of functionalities and design of information architecture	Group brainstorming; paper prototype; development from the material provided of the desired site architecture in 3 subgroups	Creation of 3 interactive PDF files (based on paper prototypes) representing what the tool should look like, what functionalities it should include, and how it should be organized
**6—Structure and skeleton: interaction design and information architecture and information design**				
	Design of information architecture (continued); interaction design (continued); design of the interface elements to facilitate the user’s interactions with the functionalities of the application: navigation, terminology, density of the text, and interface	Creation of content for different functionalities and pages and result ranking filter	Brainstorming and workshops in subgroups	Content for the caregiver profile, resource profile, needs identification questionnaire, visual analog scale, and draft of the caregiver’s testimony video and first draft of the research tool
**7—Skeleton: interface and navigation design**				
	Design of the interface elements (continued)	Validation of the content created in Session 6; discussion of privacy issues as opposed to the user experience; finalization of the algorithm database for the search tool; determination of the degree of detail of the search results; development of the keyword lexicon; and validation of the information architecture and interaction design of the site with a medium-fidelity prototype (clickable version)	Workshops in subgroups	Adjustment of the need identification questionnaire; and layout proposal for search results on the results page; draft of the keywords lexicon in association with the database
**8—Skeleton: aesthetic**				
	Graphic treatment of interface elements, and visual treatment of the text, elements on the page and navigation.	Usability test of a high-fidelity prototype (Web); presentation of different homepage proposals;-verification of the understanding of the visual analog scale; decision on which information should appear on the results page and in which form; content creation for pop-ups, pre-checking of phrases to support the quality of service, and draft for the virtual tours video; and finalization of the list of keywords (lexicon) in association within the database	Pretest; workshops in subgroups; and open discussion	Completed list of keywords (lexicon) in association with the database; collection of co-designers' impressions about the site in general; and draft of virtual tours video

### Strategy and Scope (Functionalities and Content Identification)

The first 2 CoDs, as well as the first AC, clarified the objectives of the eHealth tool and allowed for the prioritization of the needs to which it must respond (objective 1). This part of the study is described by Latulippe et al [22]. Following the identification of needs, CoDs 3, 4, 5, and 6, along with the second AC, allowed for identification of the requirements (functionalities and content) that must be included to meet the needs. Tremblay et al [23] describe this part of the project. Please also note that content elements were developed throughout the 8 CoDs. These will be presented in the *Information Design* section.

### Structure and Skeleton

**Figure 3:**
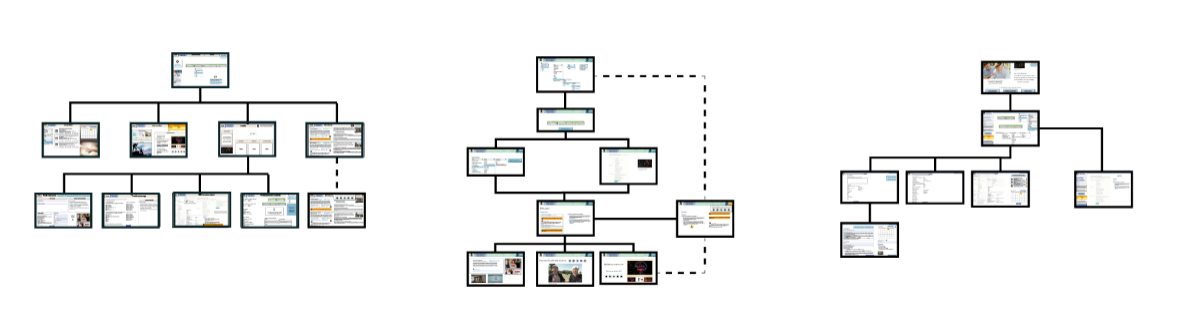
Information architecture design proposals.

The structure plane includes information architecture, which is the creation of a pattern that represents how users will access content. CoD 5 allowed for the development of the information architecture. Consequently, co-designers were divided into small working groups (including at least 1 caregiver in each). We chose low-fidelity prototyping to produce paper-based Web page designs corresponding to identified requirements. Prototyping is an effective method to advance the idea under study (here the eHealth tool) while quickly getting feedback from co-designers [17]. Thus, all functionalities and content requirements were represented by images, and co-designers were asked to place them (or remove or add them) according to the optimal organization of a website (home page, results page, etc). The 3 proposals for the information architecture design are presented in Figure 3.

Following this CoD, the research team reproduced the 3 proposed structures of the eHealth tool in an interactive PDF format. These 3 PDF proposals were then introduced to members of the advisory committee during a second meeting session.

**Figure 4:**
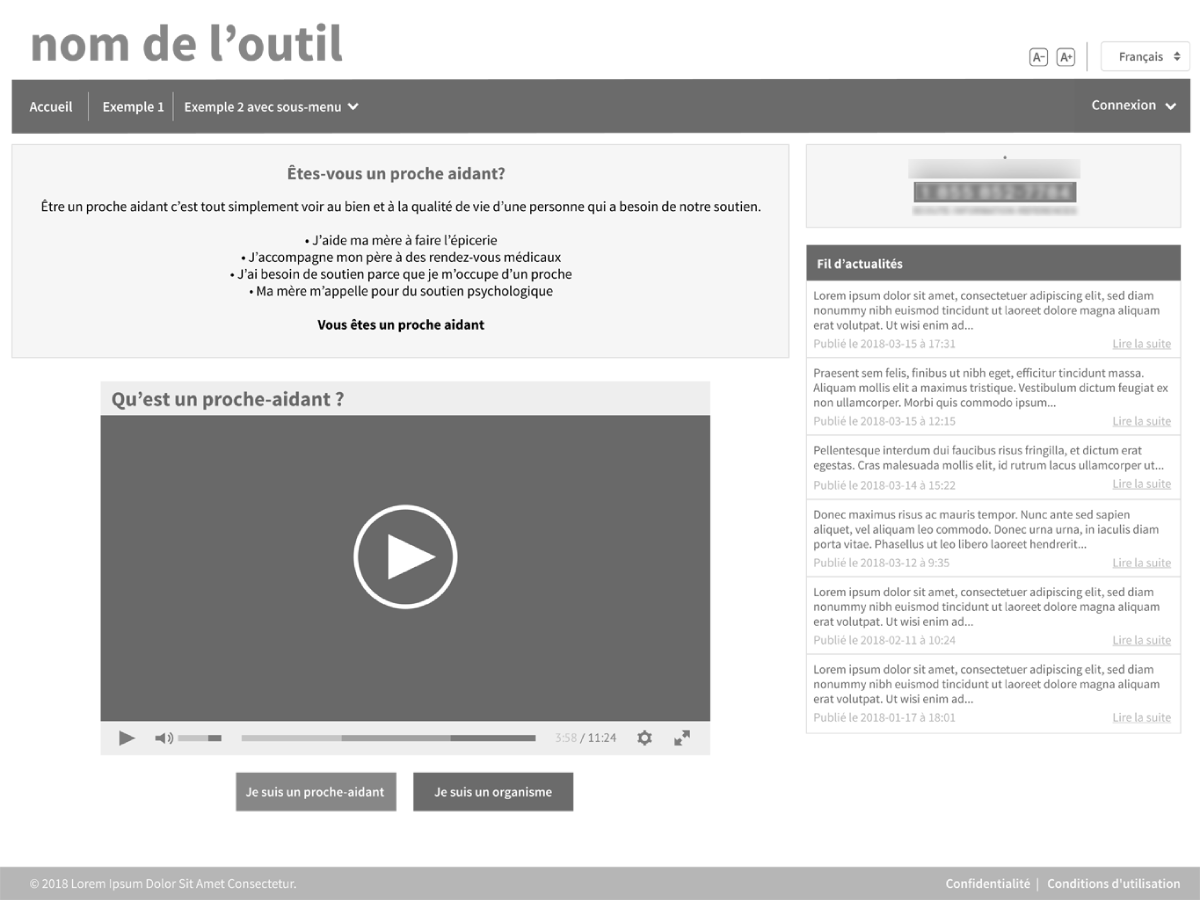
First version of the tool.

Advisory committee members were invited to explore the proposals. They were also encouraged to discuss them to make choices for the nonconsensual elements. At this stage of the project, a Web designer was recruited to design Web mock-ups, and a programmer analyst was brought in to analyze the requirements and program a first version of the eHealth tool. We deliberately chose a black-and-white version for the mock-ups of pages because we wanted to isolate aspects of information architecture, interaction, and interface design and avoid any influence that colors might have (Figure 4) [24,25].

### Information Design

**Figure 5:**
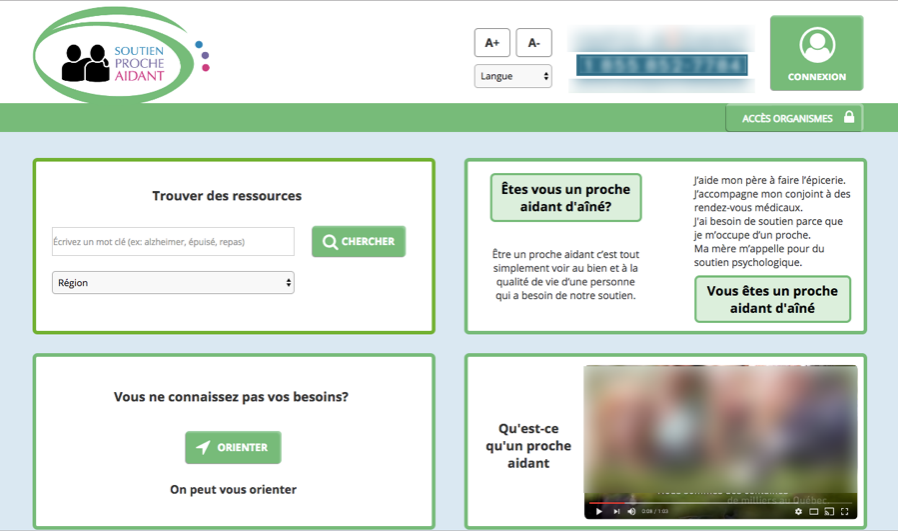
Version 2 of the tool.

We completed the production of the content items of the tool in the 6 to 8 CoDs. We also intended to work on navigation and interface elements, as well as graphic design of the tool during these sessions. However, the time required to determine the functionalities and develop the content of the tool did not allow us to complete these steps as planned. Nonetheless, even if no specific activities addressed these steps of the design process, co-designers commented on the desired colors and on the visual aspect of the tool (eg, a minimalist aesthetic), which allowed the UX expert and the programmer to develop a beta version of the eHealth tool (Figure 5).

Thus, all of these CoDs aimed to develop an eHealth tool to help caregivers in (1) recognizing their role as caregivers, (2) establishing their needs and those of the elders they support, and (3) identifying resources that meet their needs. The requirements (functionalities and content) needed to meet these objectives were identified and organized according to a structure that meets the needs and characteristics of potential users (seniors’ caregivers). In the beginning of the process, we did not know exactly the type of eHealth tool we were going to design as we let co-designers decide on the form, consistent with their needs. A website with a responsive grid that fits on a tablet was chosen because caregivers using the internet tended to use a search engine from a tablet or their computer during their help-seeking process. The website option was developed based on the actual digital literacy profile of caregivers in Quebec.

The design of the website includes the following:

1. A definition of a caregiver, including a video.
2. Reference to a resource person as needed.
3. A search tool by keywords and region or geographical area with the possibility of carrying out an advanced search.
4. A questionnaire to help caregivers identify their needs.
5. Access for organizations to register their services and activities and to submit documents and videos.
6. A Results page organizing results in 3 categories: organizations, activities, and documents.
7. The ability to add testimonials and virtual tours for each formal service to encourage caregivers and make them comfortable using the services.
8. A Profile page for caregivers where they can register their favorites and access a personal activity calendar

## Discussion

### Principal Findings

This study led to the development of an eHealth tool (a website) that allows caregivers to identify their needs and those of the older person they support and to effectively pinpoint resources to meet those needs. To date, although it is necessary to better support caregivers to preserve their health and quality of life and to ensure the safety and well-being of functionally impaired seniors, it is recognized that caregivers still have difficulty in identifying their needs and those of the elders they support and that they make scant use of available resources [6]. As there were already tools available to help caregivers in this process, co-designers first questioned the relevance of developing an additional tool. However, it emerged that none of the existing tools completely meet the needs of the caregivers or were fully adapted to their situations.

Co-design is a promising avenue for the design of eHealth technology as it has the potential to increase the correspondence between user needs and the technology developed. When users participate as co-designers, they engage as experts in terms of their own experience with technology [15]. To our knowledge, few studies have used a co-design approach to develop an eHealth tool for caregivers in Quebec. Other studies have explored a participatory approach, such as co-design in eHealth for caregivers in other countries, emphasizing the potential of including caregivers as co-designers [26-29]. This is particularly important for caregivers of older persons as they may be elderly themselves and as current statistics in Quebec reveal a digital divide related to age (65 years and more) [30]. When elder caregivers participate in the design of technologies, we can expect them to make design decisions corresponding to their willingness to use the technology designed. In this case, we believe it might increase the use of the website, enabling us to reach the goal of this research project: allowing caregivers to find appropriate resources by themselves.

### Engagement

The participation of the people targeted by the eHealth tool in the development of eHealth promotes their ability to be healthy, committed to improving the status of caregivers and, thus, reducing their risk of social health inequalities [18]. From a social justice perspective, the active participation of those concerned is a democratic process that reconciles freedoms, individual preferences, and collective choices [31]. The use of a co-design approach may allow this type of participation. It involves the groups that are experiencing the problem through a research process that combines the roles of creator, decision maker, and user simultaneously [32]. In this sense, it appears to be a genuine means of operationalizing democracy. The activities and methods used were intended to facilitate the ability of co-designer to engage in creation and innovation regardless of their technological skills.

The more concrete the methods (eg, prototyping from an image), the easier it seemed for co-designers to come up with ideas. Working in a subgroup with the presence of a moderator (a member of the team for each subgroup) encouraged a fair discussion, an ease in expressing themselves, and optimal participation for each individual.

### Challenges and Solutions

We encountered various challenges associated with the co-design approach. The principal challenges and solutions found by the research team were (1) the recruitment of caregivers, (2) discussion outside the scope of the research, (3) the limited ability of some co-designers to view functionalities, (4) the short intervals between the CoDs, (5) the place of the research team, and (6) the collaboration of experts in various domains.

#### The Recruitment of Caregivers

As with most studies using this population, the recruitment of caregivers, considering the limited time they have available, was a challenge [33]. To reduce the burden of participation and to promote recruitment, we adjusted the methodology so that caregivers were solicited for only one 3-hour work session and not for the entire co-design process. We also worked with community organizations to provide respite and transportation solutions to caregivers attending these sessions. With these adjustments, we were able to recruit 30 caregivers and achieve our objectives.

#### Discussion Outside the Scope of the Research

During the sessions, caregivers felt the need to express their emotional burden due to the role that they are fulfilling. In addition, the HSSPs and community workers wanted to communicate some frustrations related to their work. This context led toward discussions not initially planned in the working sessions. This had an impact on the time available to reach our goals. It was challenging to recognize this issue in the discussion while attempting to stay focused on the goal of the meeting. However, empathetic listening has been prioritized. We planned the sessions leaving at least 20 to 30 min without activity to have enough time to accommodate such needs. This certainly contributed to the fact that we were not able to devote a session exclusively to the sensory design stage of the Garett model, but we still managed to gather information through other activities.

#### The Limited Ability of Some Co-Designers to View Functionalities

The limited ability of some co-designers (caregivers and professionals) to view functionalities was another concern. Sometimes, co-designers were not all able to fully engage during CoDs because of a lack of design or technological knowledge or simply because of a failure to comprehend. Other studies also encountered this difficulty [28,34]. When we realized this, we explored increasingly concrete activities to facilitate this participation.

#### The Short Intervals Between the Co-Design Session

The short intervals between CoDs constituted a challenge. We planned approximately 1 CoD per month to respect our schedule. However, the time needed to analyze data and plan the following CoDs consistent with results forced us to shift some sessions. The analytical questioning method proved to be a good choice to focus on the questions to be answered for the next step. Thematic analysis, for example, would have required much more time between coding sessions.

#### The Place of the Research Team

The research team worked closely with participants in the cocreation process. This collaboration between the research team members and other co-designers might have influenced the results. That said, close interaction and collaboration between co-designers and researchers remains a fundamental aspect of the co-design approach. Knowledge creation in co-design should be considered in terms of group cognition, which includes researchers [35]. If the team is considered to be part of the co-design, it means that team members share their thoughts with other co-designers. This can influence the decisions made by the group and may compromise the group’s power sharing. Conversely, if the research team is not part of the co-design, it may have omitted some important considerations such as what is realistic for the programmer, ideas from the academic literature, or the clinical experience of members. To maintain our role as co-designers while respecting the decisions or ideas coming from other co-designers, the research team carefully noted the provenance of ideas to distinguish them from those of the other co-designers, in case there would be contradictory choices. In such a case, the advisory committee was called upon to take the decision.

#### The Collaboration of Experts in Various Domains

Another challenge stemmed from the fact that the research was conducted by experts from various domains. Therefore, a gap between the design research of insiders (those from the design domains) and that done by outsiders (researchers from other domains) emerged [36]. In fact, major difficulties in co-design are the diversity of approaches and a lack of common vocabulary to describe its characteristics, resulting in a growing bank of unrelated works and a lack of transdisciplinary understanding [14]. During the preparation sessions, we had to repeatedly clarify the vocabulary used and discuss our respective perspectives. Nevertheless, the presence of co-designers from a variety of domains has enriched the creative process and contributed to the rigor of the approach.

Thus, design is a complex cognitive activity [37], and users might encounter difficulties at certain steps of the process. Technical knowledge and technological acceptance have major impacts on design decisions. Yet, even if the investments in terms of efforts and cost might appear greatest with a multicentric and multisegment co-design user approach, it remains a promising and innovative avenue in the design of information and communication technologies in the eHealth domain. It allows for a deeper and broader understanding of human experience with technology, along with a better comprehension of nondesigners engaging in a design activity. To foster the potential efficiency of eHealth technology, we must continue to collaborate with different fields of expertise and embrace a *designerly way of thinking* when conducting co-design research. Experts from the design domain should increase collaboration with HSSPs. Design heuristics should be considered a framework for the design of eHealth technologies [38].

### Limitations

This project, beyond its challenges and solutions, has certain limitations. Among other things, the majority of participants are from the province of Quebec and speak French; we know that the notion of caregiving can vary according to different cultures [39]. In addition, the cultural competence of an eHealth tool is an important factor to consider in reducing social inequalities in health [40,41]. We were able to observe cultural differences related to the different regions (eg, feeling of strong isolation in Gaspésie, importance of the first nations in Côte-Nord, and complexity of the location of organizations in Montreal), and these differences were taken into account in the tool. However, it does not yet take into account the differences related to ethnic origin. This study will have to be continued.

### Benefits of the Project

The impacts of this project are unprecedented as it was carried out in a rigorous study involving stakeholders in 11 regions in the province of Quebec to consider the contexts that may vary according to the region. The project will definitely serve to optimize the help-seeking process through the website developed. Moreover, the questionnaire created can play in important role to support the identification of needs to assist caregivers to better prepare themselves. Even before the emergence of difficulties and depending on the trajectory of the disease, the questionnaire can support the identification of needs rather than acting in response to the gradual loss of autonomy. 

So, this not only allows for the maximizing of the autonomy, security, and quality of life of the functionally impaired older persons, but it also enables them to remain at home longer as the risk of caregiver exhaustion is reduced. This initiative will allow caregivers to have more control over various situations as they will be better equipped to cope. The benefits are also important for functionally impaired older persons as they can count on quality help from a better-equipped caregiver. Finally, throughout the project, the partnership with key players, such as members of community-based organizations and HSSPs, ensures that the proposed tool complements existing tools.

### Conclusions

This study led to the development of an eHealth tool for caregivers of functionally impaired older persons to help them identify their needs and the resources available to meet them. This tool will help caregivers to optimize their process of seeking help and to prepare for the trajectory of the disease even before the onset of hardship, rather than acting in response to an increased need for care. This proactive approach has the potential to not only maximize the autonomy, safety, and quality of life of the older person assisted but to also prolong their home care as the risk of caregiver burnout will be reduced. This initiative will allow caregivers to have more control over the various situations, as they will be better equipped to deal with them. They will also be better prepared for the evolution of the disease. Another important outcome of this project is improving the support for older persons with a loss of autonomy. Indeed, the person can count on quality help from a better-equipped caregiver. Moreover, the fact of offering this tool to caregivers as soon as the diagnosis is made will ensure elders’ right to self-determination is respected by optimizing their autonomy and involving them in decisions concerning them before difficulties arise. The next step will involve user testing to confirm the effectiveness of the design product, which will be the final stage of this study (phase 3), and an evaluation of its usability and will be done in the following months.

## Abbreviations

AC: advisory committee session
CISSS: Integrated Health and Social Service Centre
CoD: co-design session
eHealth: electronic health
HSSP: health and social service professional
PD: participatory design
PhD: Doctor of Philosophy
UX: user experience
